# Management of inflammatory bowel disease patients in the COVID-19 pandemic era: a Brazilian tertiary referral center guidance

**DOI:** 10.6061/clinics/2020/e1909

**Published:** 2020-04-13

**Authors:** Natália Sousa Freitas Queiroz, Luísa Leite Barros, Matheus Freitas Cardoso de Azevedo, Jane Oba, Carlos Walter Sobrado, Alexandre de Sousa Carlos, Luciane Reis Milani, Aytan Miranda Sipahi, Aderson Omar Mourão Cintra Damião

**Affiliations:** I Departamento de Gastroenterologia, Hospital das Clinicas HCFMUSP, Faculdade de Medicina, Universidade de Sao Paulo, Sao Paulo, SP, BR; II Divisao de Gastroenterologia e Hepatologia Clinica, Departamento de Gastroenterologia, Hospital das Clinicas HCFMUSP, Faculdade de Medicina, Universidade de Sao Paulo, Sao Paulo, SP, BR; III Instituto da Crianca e do Adolescente (ICr), Hospital das Clinicas HCFMUSP, Faculdade de Medicina, Universidade de Sao Paulo, Sao Paulo, SP, BR; IV Laboratorio de Gastroenterologia Clinica e Experimental (LIM07), Hospital das Clinicas HCFMUSP, Faculdade de Medicina, Universidade de Sao Paulo, Sao Paulo, SP, BR

**Keywords:** COVID-19, Inflammatory Bowel Disease, Coronavirus, Pandemic

## Abstract

The world is fighting the COVID-19 outbreak and health workers, including inflammatory bowel diseases specialists, have been challenged to address the specific clinical issues of their patients. We hereby summarize the current literature in the management of inflammatory bowel disease (IBD) patients during the COVID-19 pandemic era that support the rearrangement of our IBD unit and the clinical advice provided to our patients.

## INTRODUCTION

Coronavirus disease 2019 (COVID-19) is a viral respiratory illness caused by the novel coronavirus 2019 (2019-nCoV) which originated from bats 1. The 2019-nCoV is a single-stranded RNA virus and was named by the World Health Organization (WHO) as severe acute respiratory syndrome coronavirus 2 (SARS-CoV-2) because of its similarity to the virus causing severe acute respiratory syndrome. The infection outbreak began in December 2019 in Wuhan, China and rapidly spread throughout the world, including Brazil. On March 11^th^, 2020, WHO declared COVID-19 as a global pandemic. Now more than one million confirmed cases of COVID-19 have been reported to the WHO, including more than 50,000 deaths. In Brazil, as of April 4^th^, 2020, we have confirmed 10,278 infected patients and 432 deaths, and the numbers continue to increase.

COVID-19 is transmitted mainly through respiratory droplets, aerosols, and through the conjunctiva 2. The clinical spectrum of COVID-19 in adults ranges from asymptomatic infection to severe pneumonia and fatal illness. The main clinical symptoms include fever, cough, shortness of breath, myalgia, and 10-20% of patients develop acute respiratory distress syndrome after 8–14 days of the illness 3. Based on data from more than 72,000 patients from China, 81% of the patients were mildly affected, 14% had severe manifestations, and 5% were critically ill. No deaths occurred in children younger than 9 years but the mortality rate ranged from 8-15% in those aged older than 70 years 4. Risk factors for severe illness were older age and pre-existing comorbid conditions including cardiovascular disease, hypertension, diabetes, chronic respiratory disease, and cancer. Children of all ages appeared susceptible to COVID-19, but clinical manifestations were less severe compared to those seen in adults 5. Around 6% of children suffered severe disease and life-threatening conditions, but these patients commonly had a prior history of congenital or acquired disease or were younger than 1 year 4,5.

Digestive symptoms are reported in nearly half of the patients with COVID-19. Diarrhea and fever may be present in addition to respiratory symptoms 6. Since SARS-CoV-2 RNA was detected in stool samples and other clinical specimens, awareness has been raised regarding the management of patients with pre-existing digestive diseases, such as inflammatory bowel disease (IBD). Special attention was also given to potential SARS-CoV-2 transmission via a fecal route 7,8. Following viral infection, virus-specific RNA and proteins are synthesized in the cytoplasm to assemble new virions. The detection of viral RNA from feces 1 to 12 days after being negative in respiratory samples suggest that the viruses are continuously secreted from infected gastrointestinal cells 9. The period of viral secretion from pediatric feces is longer than in adults 10. These observations suggest that the inflamed gut of adults and children with IBD could be more susceptible to infection with SARS-CoV-2 because it is a doorway for the virus 11,12.

As the COVID-19 outbreak is rapidly evolving, IBD specialists from all over the world have been challenged to face the pandemic and address the specific issues of their patients, particularly regarding the risk of infection and immunosuppressive treatment. This review aims to summarize the best available evidence and expert opinion in the field of IBD and COVID-19 outbreak.

### What is the risk of COVID-19 in the IBD population?

IBD treatment often involves the prescription of immunosuppressants and IBD patients are likely to be more susceptible to infection, depending on the medications in use. As the outbreak became a rapidly spreading pandemic, several centers around the world have raised concerns that immunocompromised patients may be at increased risk of developing SARS-CoV-2 infection or severe respiratory disease 13.

In general, IBD patients taking immunomodulators may be more susceptible to infection, especially in combination with biologics, mainly anti-TNF agents. In addition, malnutrition, comorbidities, older age, previous history of serious infections, and underlying IBD activity may also be risk factors for infections 14,15.

Previous studies have demonstrated that clinically active IBD and exposure to thiopurines increase the risk of viral infections 16,17. However, unlike other viruses (Influenza, Herpes, Cytomegalovirus, Adenovirus, Rhinovirus, Norovirus, and Respiratory Syncytial Virus), coronaviruses have not been shown to cause more severe disease in immunosuppressed patients 13,15,17.

So far, there is an international consensus that patients with IBD are not at greater risk of infection with SARS-CoV-2 than the general population, although it is uncertain whether active inflammation increases the risk of getting SARS-CoV-2. It is also uncertain if IBD patients infected with SARS-CoV-2 have a higher risk of developing COVID-19 or higher rate of complications or mortality resulting from the disease, although data regarding immunosuppressed patients and SARS-CoV-2 infection are very scarce 14.

Guan et al. did not observe the use of immunomodulators as a risk factor for serious diseases in 1,099 patients in China 18. As of April 3, 2020, 12 deaths (9 male) were reported among the 275 patients with IBD and COVID-19 in the international Surveillance Epidemiology of Coronavirus Under Research Exclusion (SECURE)-IBD database. Of those, three patients were younger than 65 years old, and all received treatment with more than one immunosuppressant (steroid+JAK inhibitor; adalimumab+methotrexate; adalimumab+azathioprine+steroid) 19. Nevertheless, these cases should be interpreted with caution since many cases with mild disease were not tested for COVID-19.

In a coordinated initiative from the British Society of Gastroenterology (BSG) and Crohn's and Colitis UK association, specific guidance was provided for IBD patients according to their risk depending on the medications in use, age, and other risk factors. Through a decision tree, patients are stratified according to highest, moderate, or lowest risk of serious complications from COVID-19. Patients at the highest risk are those who either have a comorbidity (respiratory, cardiac, hypertension, or diabetes mellitus), those over 70 years old and on any immunosuppressant therapy for IBD, those of any age that are receiving ≥20 mg prednisolone, new induction therapy with biologics and immunomodulators (combination therapy) within 6 weeks, those with moderate to severe active disease or short gut syndrome, and patients requiring parenteral nutrition. Patients receiving biological therapy, thiopurines, calcineurin inhibitors, JAK inhibitors, or combination therapy are stratified as at moderate risk and the remaining IBD patients as at the lowest risk. Patients at the highest risk are required to self-isolate, while patients at moderate or lowest risk should follow strict or general populace social distancing, respectively 20.

In our center, we followed BSG recommendations regarding risk stratification, although we also considered patients subjected to IBD surgical procedures within 30 days at highest risk of serious complications of COVID-19. A brief summary of specific recommendations provided by us to the patients according to their risk is described in Table 1.

### General recommendations and strategies to minimize exposure to the virus

For the past month, emerging evidence has guided an optimized approach to prevent coronavirus infection in IBD patients to assure the safety of ongoing treatment.

Similar to the general population, IBD patients were advised to stay at home, maintain proper distance while talking, wash hands with soap or alcohol-based sanitizer, and avoid touching facial mucosa (Table 1).

Our institute, the largest in Latin America, has implemented a massive structural plan to evacuate wards, equip intensive care units, and to provide sufficient beds for patients diagnosed with COVID-19 infection. These institutional rearrangements have challenged our IBD team and a specific approach to maintain IBD care during the pandemic was needed. A summary of measures set to adapt IBD care is described in Table 2.

In the gastroenterology department, hospitalized IBD patients were reallocated to an isolated building with specialized medical staff, which has minimized exposure to the virus.

In our outpatient clinic, the great majority of appointments were rescheduled. Although a formal tele-health system was not available, medical staff closely monitored patients with severe active disease that required corticosteroids or were being treated with combination therapy via telephone calls. At this moment, laboratory tests are strictly limited, and the endoscopy unit has only been attending to urgent cases, according to the recommendations in the guidelines 11. Therefore, whenever deemed necessary, ileocolonoscopy was replaced by fecal calprotectin.

At our infusion center, the staff requested us to select patients that could have their infusion postponed for 1 or 2 weeks in order to make more space available for the rearrangement of seats. We used patients in deep clinical and endoscopic remission at the last outpatient clinic assessment as the main criteria for the rescheduling of infusions. In the course of infusions, a distance of approximately 1.5 m between seats was considered safe and no accompanying person was permitted. Although we acknowledged that the use of surgical masks for immunodeficient patients is indicated, it was not always available. Thus, a screening protocol with an objective interview was implemented prior to admission to systematically assess for acute respiratory tract symptoms, such as cough, dyspnea, or fever, among both IBD patients and their contacts. Asymptomatic patients received IV infusions as planned, while those with highly suspicious coronavirus infection were referred to the emergency room. In cases where initiation of biologics was extremely necessary, and when it was feasible, preference for those biologics that could be offered subcutaneously, at home, instead of intravenously, which would require an infusion center, was recommended.

The association of malnourishment and the risk of infections is well described in IBD literature 23,24. Hypoalbuminemia is considered a systemic inflammatory marker and is present in one-fifth of IBD patients 25,26. In the present context of the COVID-19 pandemic, social isolation could negatively impact the patient's access to healthy foods or dietary habits resulting in ensuing nutritional deficits. Thus, we have recommended a balanced diet with four to six small meals a day and adequate hydration. All patients were also encouraged to quit smoking.

In addition, IBD patients were advised to update general immunization schedules, particularly the influenza vaccination, as routinely recommended by the European Crohn's and Colitis Organization (ECCO) guidelines 27,28.

Several studies have examined the role of vitamin supplementation to control viral replication 29. Despite the lack of randomized controlled trials in this scenario, we have kept our IBD patients on vitamin D and iron replacement, if iron-deficiency anemia was detected.

### Management of IBD medications during the pandemic and potential COVID-19 infection

At the present time, it does not seem appropriate to recommend discontinuing immunosuppressant treatment in patients with IBD 11. There is a risk of reactivation of the disease for those patients who stop their treatment, and active inflammation is associated with an increased risk of hospitalization, surgery, infection, and exposure to steroids (which should be avoided in the context of COVID-19) 20,30.

The use of non-hormonal anti-inflammatory drugs should be contraindicated because of their association with adverse outcomes in other viral respiratory infections and their potential role as a trigger for the reactivation of IBD 14.

To date, there are no formal evidence-based recommendations from clinical societies or governments for immunosuppressed patients, like those with IBD. Table 3 summarizes the therapy-specific considerations for IBD patients based mainly on expert opinions and must be interpreted in a patient-specific context. These recommendations can be modified and updated as new evidence emerges 14,20.

In general, a patient with moderate to severely active Crohn's disease or ulcerative colitis should be treated with the same therapies that would be chosen, regardless of the COVID-19 pandemic 14.

In an asymptomatic IBD patient who has tested positive for SARS-CoV-2 and whose treatment has been discontinued, IBD drugs can be restarted after 14 days (assuming they have not developed COVID-19) 14.

In cases of the occurrence of signs and symptoms suggestive of COVID-19, the patient must immediately communicate with the healthcare team. In this context, it is recommended to pause immunosuppressant therapy until the infection resolves. The therapy can be resumed after complete resolution of the symptoms of COVID-19 or, ideally, after two negative PCR tests of the nasopharyngeal swab, collected with a more than 24-h interval between them (this strategy is not currently feasible in Brazil, where there is a shortage of tests for coronavirus) 14,20.

### IBD surgery in the context of the COVID-19 pandemic

The novel coronavirus has created a new dilemma for surgeons worldwide. While many patients await surgery, the risks of developing severe pulmonary infection and prolonged hospitalization are concerning. This is particularly critical for IBD patients, who are immunosuppressed by both the nature of the disease and commonly used medications, in addition to nutritional deficits. Surgical care in this context may add additional morbidity.

The main goal at this moment is to ensure the safety of patients and health care workers. Current recommendations are based largely on experts' opinions and may change rapidly as the outbreak is expected to escalate over the next weeks. A recent report from Wuhan, China, describes the outcomes of 34 patients that underwent elective surgical procedures during the COVID-19 incubation period in the early phase of the outbreak. All patients developed COVID-19 pneumonia shortly after surgery, 15 (44.1%) patients required admission to the intensive care unit and seven patients (20.5%) died 31.

Therefore, it is highly recommended by most surgical societies around the world to postpone all elective surgeries and endoscopic procedures, aiming to protect patients and surgeons and also to minimize the use of necessary resources, such as hospital beds and personal protective equipment (PPE) 32,33. Regarding the surgical procedure, there is concern for the aerosolization of the SARS-CoV-2 virus in minimally invasive procedures, although there is no robust evidence. This could also be the case with electrocautery use during open surgery. Strict use of PPE according to local practice is advisable, which includes an N95 mask and face shield protection. Negative pressure surgical rooms should be used if available and for laparoscopic surgery, a smoke evacuation system is advisable. Additionally, only essential staff should take part in the surgery.

Before embarking upon the operating room, different aspects should be considered, such as the risk of SARS-CoV-2 infection at your facility, expected length of stay, and possible surgical complications and other clinical comorbidities that may add additional morbidity/mortality. If surgical treatment is deemed necessary, informed consent should be obtained addressing the risk of SARS-CoV-2 infection. Ideally, surgical patients should be tested pre-operatively for COVID-19, although this strategy is not possible in our institution where just symptomatic patients are being tested.

There is no precise advice regarding which IBD patients would most likely benefit from surgery in this context since IBD societies around the world have not specifically addressed surgical care. Therefore, medical judgment remains essential.

Our group considers patients with anorectal septic complications (*i.e.* perianal abscess due to Crohn's disease), acute peritonitis, bowel obstruction, severe/toxic colitis (toxic megacolon), and gastrointestinal hemorrhage who fail conservative treatment are possible surgical candidates during the pandemic, but cases should be analyzed on an individual basis. Advanced colorectal malignancy that may progress to unresectable disease can also be considered for surgery. When available, minimally invasive strategies such as percutaneous drainage of an abscess may be a good strategy.

### Considerations regarding the pediatric IBD population

IBD in children often presents with more extensive and severe disease than adults. Therefore, SARS-CoV-2 infection with digestive symptoms is of particular concern in pediatric IBD (PIBD) patients. As of April 3^rd^, 2020, eleven children (younger than 19 years) have been reported to have SARS-CoV-2 infection by SECURE-IBD from a total of 275 patients. No cases were from Brazil. Only one patient was younger than 9 years; all cases were outpatients and no deaths were reported. Some questions arose: are PIBD patients at increased risk of SARS-CoV-2 infection? Is the infection in PIBD more severe than in adults? Should treatment be delayed or discontinued during the infection?

A recent paper from the Pediatric IBD PORTO Group of ESPGHAN outlined key information about children with IBD and SARS-CoV-2 infection and provided clear guidance on how to proceed 12. Chinese and Korean pediatric GI specialists reported 272 cases in children with IBD and SARS-CoV-2 infection during the pandemic while Europeans reported 8 PIBD patients (14-19 years) with COVID-19 from the PORTO-IBD group and SECURE-IBD database.

The Chinese experience reported 917 children with confirmed COVID-19 infections, and none of them had the diagnosis of IBD. In Korea, from 8,413 children with confirmed COVID-19 diagnosis, 272 (3.2%) had IBD and almost all (99.3%) continued IBD treatment. Among 4.8% of children that postponed biological infusion, 23% experienced an IBD flare.

In Europe, from the total of eight patients, seven were from the Porto-IBD and one adolescent (17 years) was from the SECURE-IBD database. The majority had Crohn's disease and most patients were in deep remission or had a moderate disease activity index, and only one had cardiovascular disease. Almost all patients had a confirmed diagnosis of COVID-19 with mild symptoms of fever, cough, fatigue, and interestingly, none of the eight patients had diarrhea.

This initial global experience may help many PIBD centers in the world implement actions during the COVID-19 pandemic. Nevertheless, many questions about children and PIBD COVID-19 infections remain unanswered. The main recommendations pertaining to treatment of PIBD during the COVID-19 pandemic are listed in Table 3.

## CONCLUSION

In the current COVID-19 pandemic, the search for safety information and guidance is critical. IBD patients have a potentially higher risk of complications of COVID-19 due to the chronic illness itself, and mainly, the use of immunosuppressant treatments. Facing these challenging circumstances, our group has adapted IBD care by resetting priorities, providing appropriate information for patients, and assuring continuity of treatments. Finally, it is of paramount importance to remember that in developing countries, a significant part of the population lives in precarious conditions, with crowding, slum dwelling, and lack of basic sanitation, among other unique features, which deserve special attention from health authorities and effective adaptations. It turns out that, in this scenario, especially in the case of IBD patients, treatment individualization is mandatory, and the benefits and disadvantages should always be taken into account.

## AUTHOR CONTRIBUTIONS

Queiroz NSF was responsible for the study conceptualization, manuscript original drafting, editing and review. Barros LL, Azevedo MFC, Sobrado CW, Carlos AS and Milani LR were responsible for manuscript drafting. Oba J was responsible for the study conceptualization, data curation, formal analysis, funding acquisition, investigation, methodology, project administration, resources planning, supervision and manuscript original drafting, editing and review. Sipahi AM and Dami�o AOMC were responsible for the manuscript editing and review.

## Figures and Tables

**Table 1:** Recommendations for IBD patients according to their risk level.

Highest risk	Moderate risk	Lowest risk
- Stay home at all times - Don't leave home to buy food, medicine or exercise - You must attend for infusions (only time you can come out) - Stay at least 2 meters (3 steps) from other people in your home whenever possible) - In case of delivery of food and medicine at your home, ask the courier to leave it outside the house - Make sure that anyone who enters your home washes their hands with soap and water for 20 seconds - Do not receive visitors, including friends and family, unless you need their help - Do not stop taking any medications without talking to your doctor	- Avoid contact with people who are showing symptoms of COVID-19 - Avoid using public transport whenever possible - Work from home whenever possible - Avoid crowds and public spaces - Avoid meetings with friends and family - Use phone services or virtual technology to contact your doctor or other essential services	For all group risks*: - Wash hands thoroughly with soap and water, for at least 20 seconds, frequently - Use 70% alcohol gel on your hands if soap and water are not available - Avoid touching eyes, nose and mouth - Clean objects and surfaces that you frequently touch (such as door handles and phones) with any cleaning product - Everyone should stay home to help prevent the spread of the virus - Avoid using public toilets+ Leave home for very limited purposes**: • Buying food and medication, • Exercise once a day, such as running, walking or cycling - alone or with a member of your family. • Donate blood or help a vulnerable person. • Travel for professional purposes, but only if strictly necessary ** Even when performing these activities, you should minimize the time spent away from home and ensure that you are 2 meters away from anyone.

* Recommendations given by the World Health Organization to the general population (21,22.)

+ The presence of viral RNA in fecal samples has been reported, thus implicating a potential route of fecal-oral transmission (19).

**Table 2:** Measures to adapt IBD care during the pandemic.

**Inpatient clinic**
- Wards of main building evacuated to provide care specifically for patients with COVID-19 - Hospitalized IBD patients relocated to an isolated building, minimizing exposure to the virus

**Outpatient clinic**
- Most visits rescheduled - Medical staff monitored patients with active disease or flare with telephone calls - Laboratory tests strictly limited - Colonoscopy replaced with fecal calprotectin

**Infusion center**
- No accompanying person permitted - Rearrangement of seats allowing a distance of approximately 1.5 m in between - Surgical masks when available - Pre-admission screening protocol to assess for acute respiratory tract symptoms among IBD patients and their contacts - Selection of patients that could have their infusion postponed for 1 or 2 week to let more space available for rearrangements of seats (deep clinical and endoscopic remission

**Table 3:** Therapy-specific considerations for inflammatory bowel disease patients (UC=ulcerative colitis; CD=Crohn’s disease).

Adults	Children
**Aminosalicylate acid derivatives (5-ASA)**	
No evidence of increased risk of COVID-19 infection. Do not stop if infected with COVID-19. Oral dose of 5-ASA should be optimized for maximum dose +/- topical (rectal), to avoid starting immunosuppressants, if possible, in patients with UC.	No evidence of increased risk of COVID-19 infection. Should never be suspended.

**Corticosteroids**	
Safety during COVID-19 infection is unclear. Corticosteroids can be used to treat disease relapses in a low dose and short period as possible. Tapering as soon as possible. Budesonide can be used for patients with ileo-caecal CD. Budesonide MMX can be used for UC patients (not available in Brazil).	Safety during COVID-19 infection is unclear. Systemic corticosteroid does not confer clinical benefit. Corticosteroids can be used to treat disease relapses in a low dose and short period as possible. Tapering as soon as possible.

Immunomodulators (Thiopurines and Methotrexate)**	
No evidence of increased risk of COVID-19 infection. Associated with the risk of serious viral infection (other than COVID-19). Initiation of monotherapy is not advised. Maintenance of combination therapy with biologics should be discussed individually. Consider stop: - Stable disease, especially when deep remission. - Elderly patients and/or those with significant comorbidities, in sustained remission. - Stop if signs and symptoms suggestive of COVID-19 develop.	No evidence of increased risk of COVID-19 infection. Immunomodulators have been prescribed without changes in doses or intervals in almost all children. - SARS-CoV2 Positive and Negative (symptomatic): Recommend suspending immunosuppressive treatment during an acute febrile illness until fever subsides and the child returns to normal health. - SARS-CoV-2 Positive (asymptomatic): Decision of therapeutic changes should be individualized.

Anti-TNF therapy*	
No evidence of increased risk of COVID-19 infection.Maintain dose and infusion interval. Consider initiation in monotherapy (adalimumab or certolizumab, as SQ, may be administered at home).Stop if develop signs and symptoms suggestive of COVID-19.	Only Infliximab and Adalimumab approved.¶No evidence of increased risk of COVID-19 infection. Maintain dose and infusion interval.# Switching from infliximab to adalimumab should be discouraged in stable patients.

Anti-IL-12/23p40 therapy (Ustekinumab)*	
No evidence of increased risk of COVID-19 infection. Monotherapy is advised. General good safety profile. Stop if develop signs and symptoms suggestive of COVID-19.	Not approved in children.

**Anti-α4β7 integrin therapy (Vedolizumab)**	
No evidence of increased risk of COVID-19 infection. Monotherapy is advised. General good safety profile. Stop if develop signs and symptoms suggestive of COVID-19.	Not approved in children.

**Janus Kinase inhibitors (tofacitinib)**	
No evidence of increased risk of COVID-19 infection however, tend to inhibit the immune response to viral infections. Initiation is not advised. Maintain therapy without increasing the dose. Stop if develop signs and symptoms suggestive of COVID-19.	Not approved in children.

* Patients who are going to start biological therapy, the subcutaneous route may be preferable on this occasion to avoid visits to clinics or hospitals.

** Stopping this therapy will not have short-term benefits, as these agents take months to clear immunosuppressing effect.

¶ Biologic plus immunomodulator in stable patients may increase risk over monotherapy but there is no specific evidence.

# Infusion facilities.

## References

[1] Zhu N, Zhang D, Wang W, Li X, Yang B, Song J (2020). A Novel Coronavirus from Patients with Pneumonia in China,
2019. N Engl J Med.

[2] Jiatong S, Lanqin L, Wenjun L (2020). COVID-19 epidemic: disease characteristics in children. J Med Virol.

[3] Huang C, Wang Y, Li X, Ren L, Zhao J, Hu Y (2020). Clinical features of patients infected with 2019 novel coronavirus in
Wuhan, China. Lancet.

[4] Wu Z, McGoogan JM (2020). Characteristics of and Important Lessons From the Coronavirus Disease 2019
(COVID-19) Outbreak in China: Summary of a Report of 72314 Cases From the Chinese Center
for Disease Control and Prevention. JAMA.

[5] Dong Y, Mo X, Hu Y, Qi X, Jiang F, Jiang Z (2020). Epidemiological Characteristics of 2143 Pediatric Patients With 2019
Coronavirus Disease in China. Pediatrics.

[6] Lin L, Jiang X, Zhang Z, Huang S, Zhang Z, Fang Z (2020). Gastrointestinal symptoms of 95 cases with SARS-CoV-2
infection. Gut.

[7] Mao R, Liang J, Shen J, Ghosh S, Zhu LR, Yang H (2020). Implications of COVID-19 for patients with pre-existing digestive
diseases. Lancet Gastroenterol Hepatol.

[8] Wang W, Xu Y, Gao R, Lu R, Han K, Wu G (2020). Detection of SARS-CoV-2 in Different Types of Clinical
Specimens. JAMA.

[9] Xiao F, Tang M, Zheng X, Liu Y, Li X, Shan H (2020). Evidence for Gastrointestinal Infection of SARS-CoV-2. Gastroenterology.

[10] Ma X, Su L, Zhang Y, Zhang X, Gai Z, Zhang Z (2020). Do children need a longer time to shed SARS-CoV-2 in stool than
adults?. J Microbiol Immunol Infect.

[11] Danese S, Cecconi M, Spinelli A (2020). Management of IBD during the COVID-19 outbreak: resetting clinical
priorities. Nat Rev Gastroenterol Hepatol.

[12] Turner D, Huang Y, Martin-de-Carpi J, Aloi M, Focht G, Kang B (2020). COVID-19 and Paediatric Inflammatory Bowel Diseases: Global Experience and
Provisional Guidance (March 2020) from the Paediatric IBD Porto group of
ESPGHAN. J Pediatr Gastroenterol Nutr.

[13] D'Antiga L (2020). Coronaviruses and immunosuppressed patients. The facts during the third
epidemic. Liver Transpl.

[14] Rubin DT, Abreu MT, Rai V, Siegel CA Management of Patients with Crohn’s Disease and Ulcerative Colitis
During the COVID-19 Pandemic: Results of an International Meeting. Gastroenterology.

[15] Rahier JF, Magro F, Abreu C, Armuzzi A, Ben-Horin S, Chowers Y (2014). Second European evidence-based consensus on the prevention, diagnosis and
management of opportunistic infections in inflammatory bowel disease. J Crohns Colitis.

[16] Kirchgesner J, Lemaitre M, Carrat F, Zureik M, Carbonnel F, Dray-Spira R (2018). Risk of Serious and Opportunistic Infections Associated With Treatment of
Inflammatory Bowel Diseases. Gastroenterology.

[17] Wisniewski A, Kirchgesner J, Seksik P, Landman C, Bourrier A, Nion-Larmurier I (2019). Increased incidence of systemic serious viral infections in patients with
inflammatory bowel disease associates with active disease and use of
thiopurines. United Eur Gastroenterol J.

[18] Guan WJ, Ni ZY, Hu Y, Liang WH, Ou CQ, He JX (2020). Clinical Characteristics of Coronavirus Disease 2019 in
China. N Engl J Med.

[19] Hindson J (2020). COVID-19: faecal-oral transmission?. Nat Rev Gastroenterol Hepatol.

[20] British Society of Gastroenterology (BSG) (2020). British Society of Gastroenterology (BSG) advice for management of
inflammatory bowel diseases during the COVID-19 pandemic.

[21] Swan C, Duroudier NP, Campbell E, Zaitoun A, Hastings M, Dukes GE (2013). Identifying and testing candidate genetic polymorphisms in the irritable
bowel syndrome (IBS): association with TNFSF15 and TNFα. Gut.

[22] World Health Organization Q&A on coronaviruses (COVID-19)
[Internet] (2020). https://www.who.int/news-room/q-a-detail/q-a-coronaviruses.

[23] Massironi S, Rossi RE, Cavalcoli FA, Della Valle S, Fraquelli M, Conte D (2013). Nutritional deficiencies in inflammatory bowel disease: therapeutic
approaches. Clin Nutr.

[24] Soeters PB, Wolfe RR, Shenkin A (2019). Hypoalbuminemia: Pathogenesis and Clinical Significance. JPEN J Parenter Enteral Nutr.

[25] Irwin JR, Ferguson E, Simms LA, Hanigan K, Doecke JD, Langguth D (2019). Detectable Laboratory Abnormality Is Present up to 12 Months Prior to
Diagnosis in Patients with Crohn's Disease. Dig Dis Sci.

[26] Nguyen GC, Du L, Chong RY, Jackson TD (2019). Hypoalbuminaemia and Postoperative Outcomes in Inflammatory Bowel Disease:
the NSQIP Surgical Cohort. J Crohns Colitis.

[27] Torres J, Bonovas S, Doherty G, Kucharzik T, Gisbert JP, Raine T (2020). ECCO Guidelines on Therapeutics in Crohn's Disease: Medical
Treatment. J Crohns Colitis.

[28] Harbord M, Eliakim R, Bettenworth D, Karmiris K, Katsanos K, Kopylov U (2017). Third European Evidence-based Consensus on Diagnosis and Management of
Ulcerative Colitis. Part 2: Current Management. J Crohns Colitis.

[29] Zhang L, Liu Y (2020). Potential interventions for novel coronavirus in China: A systematic
review. J Med Virol.

[30] Lamb CA, Kennedy NA, Raine T, Hendy PA, Smith PJ, Limdi JK (2019). British Society of Gastroenterology consensus guidelines on the management
of inflammatory bowel disease in adults. Gut.

[31] Lei S, Jiang F, Su W, Chen C, Chen J, Mei W (2020). Clinical characteristics and outcomes of patients undergoing surgeries
during the incubation period of COVID-19 infection. EClinicalMedicine.

[32] SAGES and EAES recommendations regarding surgical response to COVID-19
crisis (2020). http://www.sages.org/recommendations-surgical-response-covid-19/.

[33] Posicionamento do CBCD quanto ao COVID-19 (2020). http://www.cbcd.org.br/cbcdnews/2020/posicionamento-do-cbcd-quanto-ao-covid-19/.

